# A Rare Cause of Chronic Hypokalemia with Metabolic Alkalosis: Case Report and Differential Diagnosis

**DOI:** 10.3390/children7110212

**Published:** 2020-11-05

**Authors:** Cristina Bertulli, Marguerite Hureaux, Chiara De Mutiis, Andrea Pasini, Detlef Bockenhauer, Rosa Vargas-Poussou, Claudio La Scola

**Affiliations:** 1 Nephrology and Dialysis Unit, Department of Pediatrics, Azienda Ospedaliero Universitaria Sant’Orsola-Malpighi, 40138 Bologna, Italy; cristina.bertulli@aosp.bo.it (C.B.); chiarademutiis@yahoo.com (C.D.M.); andrea.pasini@aosp.bo.it (A.P.); 2 Assistance Publique Hôpitaux de Paris, Department of Genetics, Hôpital Européen Georges-Pompidou, 75015 Paris, France; marguerite.hureaux@aphp.fr (M.H.); rosa.vargas@aphp.fr (R.V.-P.); 3 Department of Paediatric Nephrology, Great Ormond Street Hospital for Children NHS Foundation Trust, London WC1N 3JH, UK; d.bockenhauer@ucl.ac.uk; 4 Department of Renal Medicine, University College London, London WC1E 6BT, UK

**Keywords:** hypokalemia, metabolic alkalosis, renin, aldosterone, monogenic hypertension, apparent excess of mineralcorticoid

## Abstract

Hypokalemia and metabolic alkalosis can be present in different rare diseases, and the differential diagnosis of these forms is challenging. Apparent mineralcorticoid (AME) excess syndrome is one of these conditions. Characterized by increased blood pressure due to excessive sodium retention and plasma volume, it is caused by a mutation in the *HSD11B2* gene encoding the oxydoreductase enzyme 11β-hydroxysteroide dehydrogenase type 2. We report the case of a child presenting with failure to thrive associated with early detection of hypokalemia, metabolic alkalosis, nephrocalcinosis and hypertension in which AME syndrome was detected. A novel mutation in the *HSD11B2* gene was identified in this patient. In clinical pictures characterized by metabolic alkalosis and hypokalemia, the evaluation of renin, aldosterone and blood pressure is crucial for accurate diagnosis. AME syndrome is a rare disorder that can be an insidious but lethal disease, if untreated. With clinical signs appearing during the first days of life. Early diagnosis is imperative in order to enable prompt and adequate treatment to improve the outcome of these patients.

## 1. Introduction

**Figure 1:**
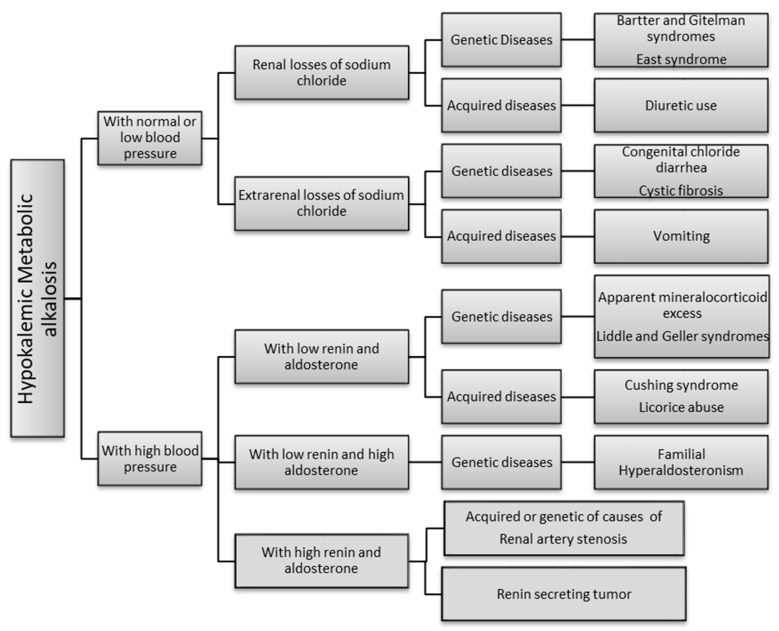
Algorithm of the causes of hypokaliemic metabolic alkalosis. Most of these disorders are rare, with a potentially insidious clinical presentation and diagnosis is often delayed or missed [7,8]. In addition, age at onset can be variable, with the most severe forms usually being detected in early childhood.

Hypokalemic metabolic alkalosis can result from two different mechanisms: (1) the renal loss of salt associated with low or normal blood pressure and secondary hyperaldosteronism (Bartter and Gitelman syndrome, East Syndrome, use of diuretics) or the extrarenal loss of salt (congenital chloride diarrhea, cystic fibrosis, vomiting); (2) an increase in sodium reabsorption and potassium secretion not related to direct aldosterone action, associated with high blood pressure. In these cases, renin and aldosterone can be both low (Apparent mineralcorticoid excess, Liddle and Geller syndrome, Cushing syndrome, licorice abuse), renin low and aldosterone high (familial hyperaldosteronism) or can be both high (renal artery stenosis, renin secreting tumors). From a clinical point of view, these two mechanisms can be differentiated by blood pressure measurement. Figure 1 summarizes the genetic and acquired diseases associated with these two main etiologies [1,2,3,4,5,6].

Apparent mineralcorticoid excess (AME) (OMIM# 218030) is one of the most severe diseases associated with hypokalemia and metabolic alkalosis. The syndrome can occur in two forms. Type 1 is the more severe form with onset in infancy, while Type II is a milder form generally diagnosed in adulthood. In its most severe form, AME causes low birth weight, failure to thrive, severe hypertension with end organ damage that can involve the renal, neurological, neuromuscular, cardiovascular and ocular systems and which may, in some cases, lead to early death in the absence of treatment [7,9,10]. Apparent mineralcorticoid excess syndrome is caused by a mutation in the *HSD11B2* gene [11]. The gene is located on chromosome 16 (16q22.1), encoding the oxydoreductase enzyme 11β-hydroxysteroide dehydrogenase type 2 (11βHSD2), mainly expressed in the collecting duct of the nephron and in the colonic epithelium [12]. It is also highly expressed in the placenta, likely explaining the associated intrauterine growth retardation (IUGR). The mineralocorticoid receptor is not specific for mineralocorticoids but can also be activated by cortisol, but not cortisone. As plasma cortisol concentrations are 100- to 1000-fold higher than plasma aldosterone, 11βHSD2 plays a crucial role by converting cortisol into cortisone in the mineralocorticoid target cells, thereby preventing inappropriate activation of the mineralocorticoid receptor.

We report the case of a child with AME affected by hypokalemia and metabolic alkalosis since birth, and we discuss the steps of differential diagnosis.

## 2. Case Presentation

We report the case of a second-born female child to consanguineous Pakistani parents (first degree cousins); both parents and the sibling are in good health. The baby was born at 34 weeks of gestation and perinatal medical history revealed intrauterine growth retardation and oligohydramnios. At birth, she was small for gestational age (weight 1420 g—4.98 SDS, length 39 cm—5.45 SDS) and was admitted to the neonatology ward for feeding difficulties. Laboratory tests (Table 1) showed a mild hypokalemia with metabolic alkalosis, which was not investigated further. She was subsequently discharged at 4 days of life in good overall clinical condition, and no therapy was started.

**Figure 2:**
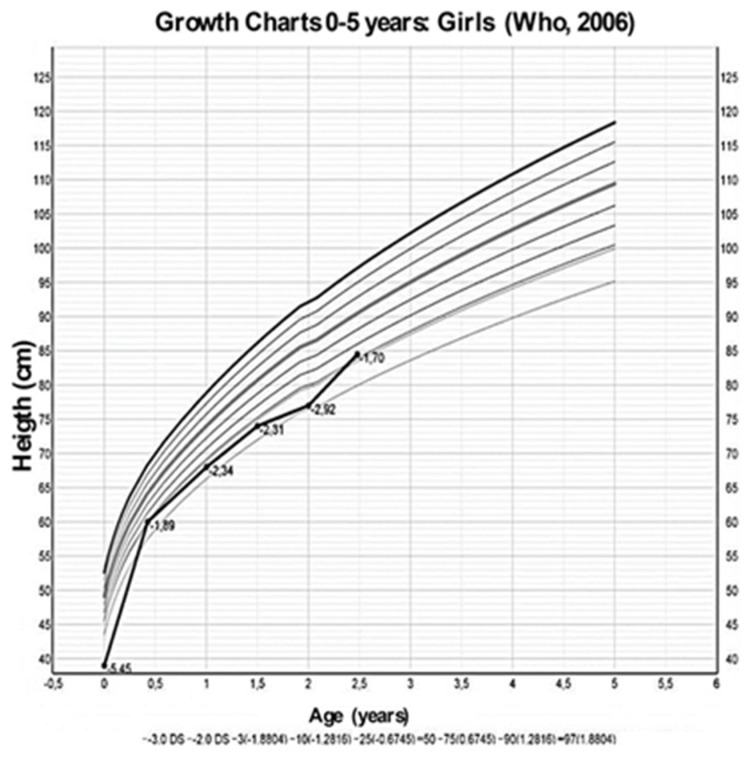
Growth charts, from Who 2006 [13], of the height of the patient at different ages.

**Figure 3:**
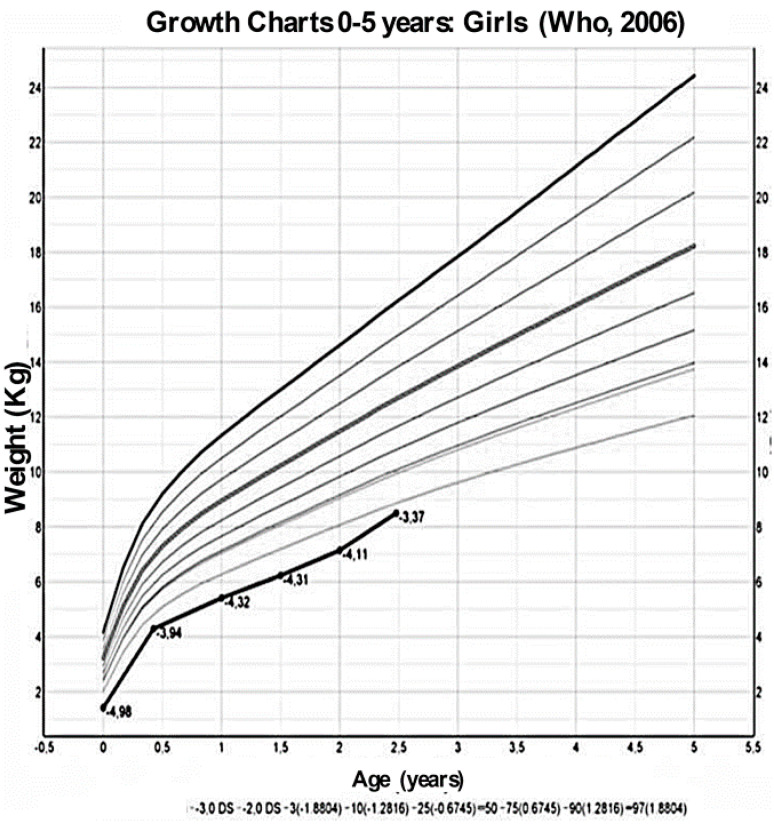
Growth charts, from Who 2006 [13], of the weight of the patient at different ages.

At 7 months of age, due to her failure-to-thrive (Figure 2 and Figure 3), laboratory tests were repeated, and mild hypokalemia with metabolic alkalosis was confirmed, this time associated with low renin and aldosterone values, which had not been tested for previously.

An echocardiographic examination yielded normal results, while a renal ultrasound raised the suspicion of nephrocalcinosis, with normal urinary calcium excretion; other urinary electrolytes were not analyzed. Due to the association of hypokalemia, nephrocalcinosis and failure to thrive, a classic Bartter syndrome type III was suspected, despite the low renin and aldosterone values and the presence of nephrocalcinosis, not confirmed by genetic testing. Blood pressure measurements were not evaluated.

**Figure 4:**
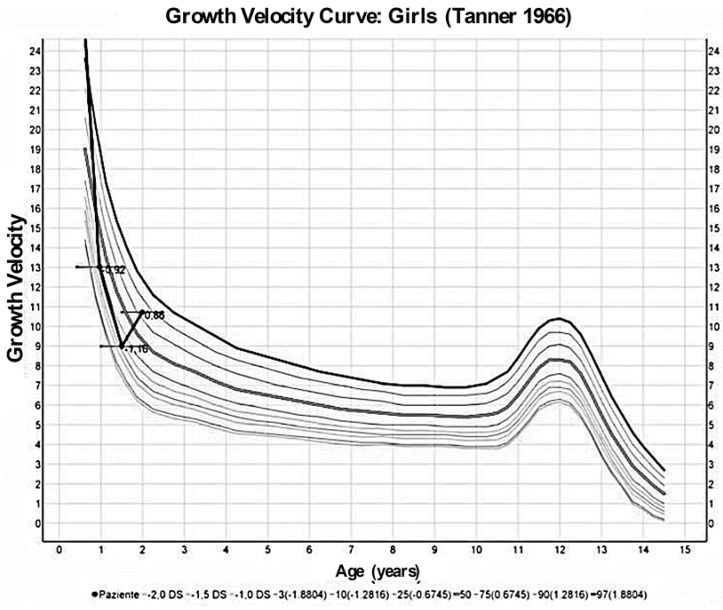
Growth velocity curve by Tanner 1966 [14] of the patient at different ages.

At 9 months of age, the child was admitted to a pediatric nephrology unit at a tertiary level hospital for persistent growth failure (Figure 2, Figure 3 and Figure 4); weight and height were 4740 g (−4.7 SDS) and 63 cm (−3.0 SDS), respectively.

Mild polyuria with a urine output of 106 mL/kg/day and low urine osmolality (105 mOsm/kg) were observed. The ratio between urinary potassium and urinary creatinine was high (uK/uCr 17 mmol/mmol), while the fractional excretion of sodium was below 1% (FENa 0.7%) (Table 1). A new echocardiographic examination revealed mild ventricular septal hypertrophy (thickness of interventricular septum at end diastole IVSD 6.3 mm), which was referred to follow up after six months; blood pressure readings taken daily over the four-day hospital stay were occasionally very high but otherwise within normal ranges. During the following months, the polyuria worsened (250 mL/kg/day), urinary calcium levels increased (Table 1), and supine and standing blood pressure values were consistently elevated (>99th percentile for sex, age, and height). Echocardiographic examination at 15 months showed ventricular hypertrophy (IVSD 7–8 mm), which led to the introduction of antihypertensive therapy with calcium antagonists and beta-blockers, with little benefit.

High blood pressure values associated with hypokalemia and low renin and aldosterone levels were suggestive of a monogenic form of hypertension. Taking into account the antecedent of parental consanguinity and the age at presentation, Liddle and Geller syndromes were excluded. Congenital adrenal hyperpeplasia types IV and V had been excluded by routine neonatal screening performed during the first days of life. Moreover, subsequent laboratory tests showed normal deoxycorticosterone, corticosterone, 18-hydroxydeoxycorticosterone, and 18-hydroxycortisol levels. With these data, the most likely diagnosis was AME Syndrome, which was confirmed by 24 h urine steroid metabolome analysis that showed the typical constellation of 11β-hydroxysteroid dehydrogenase type 2 (11βHSD2) deficiency with an elevated ratio of tetrahydrocortisol (THF) + allo-tetrahydrocortisol (5α-THF) to tetrahydrocortisone (THE) (ratio of 20 for a normal range of 0.75–1.50).

**Figure 5:**
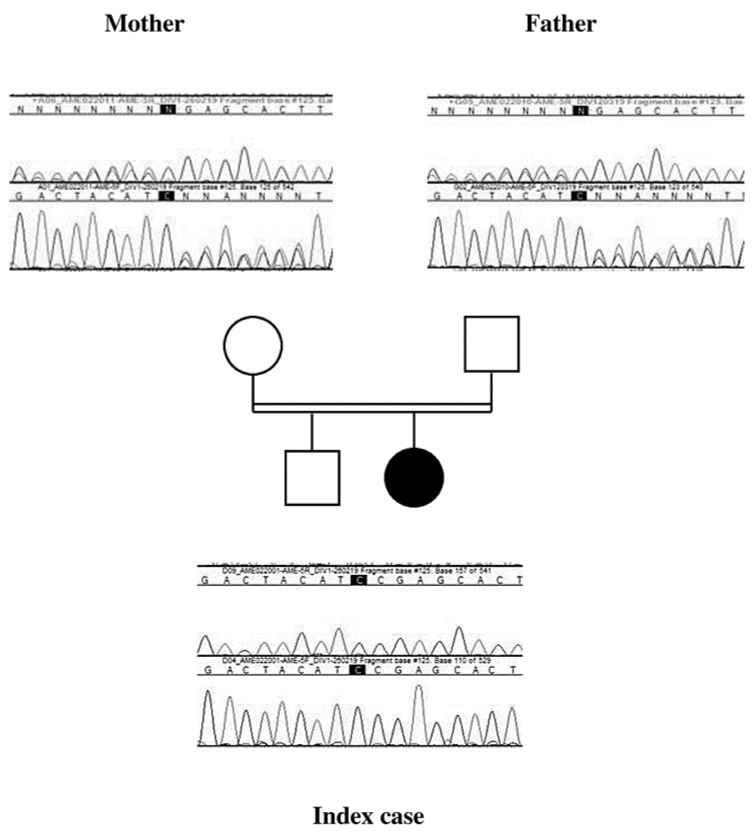
The chromatographic profile of each parent shows a superposition of the genomic sequences of the two alleles, one allele wild-type and the second with the mutation at position c.900, confirming the heterozygous status. Index case chromatogram shows only one genomic sequence, confirming the homozygous status for the pathogenic variant c.[900dup];[900dup];p.[(Glu301Argfs*56); (Glu301Argfs*56)].

Direct sequencing of the *HSD11B2* gene by Sanger revealed a homozygous frameshift variant in exon 5, c.900 dup, p. Glu301Argfs*56. Analysis of the parents showed that both were heterozygous carriers of the variant, consistent with their consanguinity (Figure 5).

This previously undescribed variant was considered pathogenic (class 5, ACMG 2015 classification) [15]. Criteria used to classify this variant are given in Supplementary Table S1. To date, 44 different mutations has been described in the *HSD11B2* gene in the HGMD professional database (http://www.hgmd.cf.ac.uk/ac/all.php). They are scattered in all the 5 exons of the gene, and most of them are missense mutations (61%), followed by in-frame deletions or insertions (16%) and mutations introducing a premature stop codon (nonsense and frameshift mutations: 14%).

A low sodium diet was started and spironolactone (1 mg/kg/day) and amiloride (200 microgr/kg/day) were prescribed. Three months later, blood pressure values had improved with values ranging between 50–90th percentiles, and electrolytes and blood pH had normalized; at 30 months of life, urine osmolality had increased to 350 mOsm/kg (Table 1) though mild polyuria persisted, growth velocity had changed from −0.92 SDS at diagnosis to +0.86 SDS (Figure 4) while her weight, even if increased, remained below −3 SD. Finally, a new echocardiographic examination at 36 months of life showed regression of the ventricular hypertrophy, while nephrocalcinosis at renal US was unchanged.

Treatment and data gathering was conducted according to local law and the parents gave consent to participate on behalf of the patient. Full informed consent for gathering the data and for publication of this case report was given by the parents on behalf of the patient.

## 3. Discussion

We report the case of an infant with AME syndrome which was initially misdiagnosed as Bartter syndrome III type, despite low renin and aldosterone levels. Recently, Najafi et al. described a cohort of 17 children presenting with hypokalemic metabolic alkalosis, low birth weight and failure to thrive, 4 of them were clinically misdiagnosed as having Bartter Syndrome. Using Whole Exome Sequencing, different diagnoses were discovered in 4 of these patients including cystic fibrosis, congenital chloride diarrhea and AME [8]. In their patients, as in ours, diagnosis was delayed because blood pressure monitoring had not been routinely performed, highlighting the difficulties of getting accurate blood pressure measurements in small infants. Nevertheless, even when hypertension is detected, a diagnosis of AME can be delayed for many years [7,8,9]. For example, Morineau et al. reported a mean age at clinical diagnosis of hypertension of about 5 years of life in a cohort of patients with AME, while in the same cohort the mean age at diagnosis of AME was approximately 15 years [7].

Evaluation of the urinary electrolytes, renin-angiotensin-aldosterone system (RAAS), and accurate blood pressure monitoring are important steps in the differential diagnosis of patients with persistent hypokalemia and metabolic alkalosis [1]. The salt-wasting inherited disorders like Bartter and Gitelman syndromes are associated with extracellular fluid volume depletion, low or normal blood pressure and an increase in RAAS activity. Conversely, high blood pressure with low renin activity is present in monogenic forms of hypertension as a consequence of increased sodium reabsorption in the distal nephron, with subsequent extracellular volume expansion. The spectrum of monogenic hypertension with hypokalemia includes disorders with low renin activity that can be inherited in an autosomal or recessive way [4,5,16]. Most of these conditions also show metabolic alkalosis, and aldosterone levels can be variable: low or normal in AME, Liddle, congenital adrenal hyperplasia (CAH), and Geller syndrome, and high in Familial Hyperaldosteronisms (Table 2).

The cornerstone of the pathogenesis of these inherited low-renin forms of hypertension with hypokalemia is the increase in sodium reabsorption in the distal tubule. Different mechanisms can produce this increase: (1) gain-of-function mutations in the epithelial sodium channel (e.g., in Liddle Syndrome) or the mineral corticoid receptor (e.g., in Geller Syndrome), (2) mutations in genes encoding for enzymes that regulate adrenal steroid metabolism and activity (e.g., AME syndrome and CAH). Nevertheless, it is important to note that the FENa in our infant was normal at presentation at 0.7%. This is because in steady state, urinary sodium excretion must be equal to sodium intake. A persistently lower sodium excretion would lead to progressive volume expansion with subsequent death from stroke or cardiac failure. The high mineralocorticoid activity in AME will lead to initial salt retention and volume expansion with consequent upregulation of compensatory mechanism, such as pressure natriuresis, until a new steady state at an increased circulating volume is established. This mirrors the findings in salt-wasting conditions, where a compensatory mechanism leads to a new steady state at decreased circulating volume. FENa at steady state is thus not a useful diagnostic tool [3].

An interesting observation is also the polyuria with low urine osmolality, which improves, as blood pressure and electrolytes normalize. This observation has been previously described in AME as a reversible secondary form of nephrogenic diabetes insipidus [17].

About 100 cases of AME syndrome have been described to date, most of them in consanguineous and endogamous populations due to the autosomal recessive transmission [9]. Generally, in type I cases, diagnosis is made after the second year of life [7,9,17]. In a recent report on a cohort of 36 patients with AME, Yau et al. reported a median age at diagnosis of 4.6 years (range: 0.1–15) [9]. Most of the previously reported cases of AME lack well-documented clinical, laboratory and radiological findings in the first months of life, even in patients whose diagnosis is made during the first year of life. Thus, the point at which the typical findings of AME appear is debated; some authors have stated that alterations like electrolyte disturbances and hypertension are absent in early life even though AME is a congenital disease [18].

However, the clinical presentation of our patient demonstrates that hypokalemia and metabolic alkalosis can be present at birth and that growth delay can occur also before birth with IUGR. Data from other authors show a variable age at onset/diagnosis of hypertension, in most cases in childhood and adolescence [7,8,9]. Unfortunately, in our case, the exact time of onset of hypertension is unknown because blood pressure readings were not taken until the patient was nine months old, when she was referred to our unit. However, if we consider the normal echocardiography at 7 months of life and the detection of hyporeninemia, a marker of volume expansion, and then the discovery of left ventricular hypertrophy two months later, it is not unreasonable to speculate that the onset of severe and sustained hypertension occurred between 7 and 9 months of age. Left ventricular hypertrophy is present in some patients, probably those with the most severe form of hypertension, but not in all, and it can lead to early death if hypertension is left untreated [7].

As regards the prenatal history of our patient, IUGR and oligohydramnios were present. A mild to moderate degree of IUGR has been reported in the literature, probably due to the fact that a deficiency in 11βHSD2 in the placenta permits excessive quantities of maternal glucocorticoids to cross the placenta and thus inhibits fetal growth [10,19,20].

The presence of oligohydramnios in the prenatal period is more difficult to explain considering the fact that AME is a polyuric disorder associated to low urine osmolality. The mechanism of polyuria is not well known but could most likely be due to nephrogenic diabetes insipidus induced by chronic hypokalemia. In our patient, polyuria seemed to appear and worsen during the course of the first year of life, despite the fact that hypokalemia was mild [10,17].

Another important feature of the classic form of AME, as seen in our case, is the presence of nephrocalcinosis with very mild hypercalciuria. The mechanism of these alterations is also unclear, and some authors hypothesize a correlation with chronic long-standing hypokalemia [21]. However, in our patient, nephrocalcinosis persisted despite the normalization of potassium levels.

The early onset of AME in our patient, together with the severity of the clinical spectrum, can be explained from both a biochemical and genetic point of view, given the mutation found.

The urinary cortisol to cortisone ratio directly reflects the renal 11HSB2 activity and it is inversely correlated with age at diagnosis [22,23]. Morineau et al. described the relationship between the in vitro and the in vivo activity of 11βHSD2; when no residual activity of the expressed mutation was detectable in vitro, all the affected patients showed relevant decreased 11βHSD2 activity in vivo, corresponding to a urinary ratio of THF+5α-THF/THE values over 20, as seen in our patient, and in patients with severe and early clinical presentation [7,11].

Truncating mutations (nonsense, frameshift) can lead to dysfunction or total absence of the protein, with no or very reduced activity [24]. Indeed, the presence of premature STOP codon results in early degradation of the mRNA by the nonsense mediated mRNA decay (NMD) process, thus preventing the translation of a truncated potentially deleterious protein [25]. On the other hand, the consequences of missense mutations or in-frame deletions/insertions (including splice abnormalities) are less certain. For this reason, Yau et al. developed an in silico molecular model of the 11βHSD2 enzyme and studied the genotype-phenotype correlation for missense mutations, in order to deduce the severity of the phenotype according to the mutation location [9]. As the active form of 11βHSD2 is the monomer, they showed that missense mutations that promote dimerization, alter substrate or coenzyme binding, or impair the structural stability of 11βHSD2 yield severe AME [26,27]. Previously to these results, reduced protein stability rather than reduced catalysis was shown to be the cause of reduced 11βHSD2 activity for COOH-terminal truncated forms with rapid protein degradation at the proteasome [12]. In vitro studies of a frameshift mutation showed a total absence of enzyme activity, associated with the severe phenotype [26]. Truncated proteins were also found in patients with stillbirth [12,28]. Our patient harbors a frameshift mutation, which may explain her early and severe clinical presentation.

## 4. Conclusions

Our case demonstrates that, in contrast to the cases previously described in the literature, certain signs and symptoms such as hypokalemia and metabolic alkalosis can be present from the first days of life. It is very difficult to make a differential diagnosis in these very rare cases presenting with hypokalemia and alkalosis, so evaluation of the RAAS and blood pressure is always important. The early diagnosis of hypertension allows for the timely prescription of adequate treatment and helps reduce the development and progression of organ damage. When the clinical diagnosis is ambiguous or para-clinical investigations are not available, a genetic diagnosis with a panel of genes including the known genes causing hypokalemia with metabolic alkalosis could be helpful.

## Figures and Tables

**Table 1:** Laboratory exams of patient at different ages.

FE	4 dd	7 mo	9 mo	12 mo	15 mo	18 mo	30 mo	36 mo	
**Creatinine**	0.2	0.3	0.26	0.3	0.3	0.3	0.3	0.3	mg/dL
**Na**	137	138	140	139	140	139	139	139	mmol/L
**K**	3.3	3.3	3.1	3.3	3.3	3.6	4.2	4.6	mmol/L
**Cl**	101	102	101	102	101	101	101	100	mmol/L
**pH**	7.51	7.51	7.51	7.47	7.45	7.37	7.37	7.37	
**HCO^3−^**	27	27	31.1	33.5	31	24	28.3	28.3	mmol/L
**Renine**	−	2.1	1.8	1.7	1.8	−	3.6	−	pg/mL
**Aldosterone**	−	3.6	3.6	3.6	3.6	−	2.6	−	microU/mL
**CaU/CrU**	−	<0.2	<0.2	0.5	0.8	0.5	0.5	0.5	mg/mg
**FENa**	−	−	0.7	−	−	0.22	0.26	-	%
**uK/uCr**	−	−	17	−	−	31	27	−	mmol/mmol
**OSM U**	−	−	130	−	132	236	350		mOsm/L
**Renal ultrasound**	−	Nephrocalcinosis	−	−	−	−	−	Nephrocalcinosis	
**Cardiac ultrasound**	−	Normal	Mild ventricular septal hypertrophy (IVSD 6.3 mm)	−	Ventricular hypertrophy (IVSD 7–8 mm)	−	−	Normal	
**Therapies**	−	None	None	None	Carvedilol Amlodipine	Carvedilol Amlodipine	Spironolactone Amiloride Amlodipine	Spironolactone Amiloride Amlodipine	

dd: days; mo: months; FENa: fractional excretion of sodium.

**Table 2:** Genetic and acquired diseases associated with hypokalemic metabolic alkalosis.

	Bartter	Gitelman	Liddle	AME	Geller	Congenital Adrenal Hyperplasia	Familial Hyperaldosteronism
**Potassium**	Low	Low	Low	Low	Low	Low	Normal or Low
**Sodium Bicarbonate**	High	High	High	High	High	High	High
**Renin**	High	High	Low	Low	Low	Low	Low
**Aldosterone**	High	High	Low	Low	Low	Low	High
**Hypertension**	No	No	Yes	Yes	Yes	Yes	Yes
**Cortisol/ACTH**	−	−	−	N/N	−	Low/High	
**Age of onset**	Infancy	Childhood/Adulthood	Childhood/Adulthood	Every age	Adulthood	Infancy	Childhood/Adulthood
**Transmission**	AD-AR	AR	AD	AR	AD	AD	AD
**Alteration**	NaKCl cotransporter 2 Renal outer medullary K channel Cl channel Barttin Ca sensing receptor	NaCl cotransporter Cl channel Kb	Epithelial Na channel gain of function	Defect in11-beta-hydroxysteroid dehydrogenase type 2	Gain of function mutation of mineralocorticoid receptor	Steroid synthesis defect with gain of function of mineralocorticoid receptor	Increased levels of 18-oxocortisol and 18-hydroxycortisol
**Gene**	SLC12A1 KCNJ1 CLCNKB BSND CASR	SLC12A3 CLCNKB	SCNN1B SCNN1G	HSD11B2	NR3C2	CYP21A2	CYP11B1/CYP11B2 CLCN2 KCNJ5 CACNA1H

AME: apparent excess of mineralcorticoid; ACTH: adreno cortico tropin hormone; N: normal; AR: autosomic recessive; AD: autosomic dominant.

## Supplementary Materials

The following are available online at https://www.mdpi.com/2227-9067/7/11/212/s1. Table S1: Criteria for Classifying Pathogenic Variants according to ACMG guidelines.

## Abbreviations

AME: Apparent Excess of Mineralcorticoid; 11βHSD2: 11β-hydroxysteroid dehydrogenase type 2; THF: tetrahydrocortisol; 5α-THF: allo-tetrahydrocortisol; THE: tetrahydrocortisone; RAAS: renin-angiotensine-aldosterone system; IUGR: intrauterine growth retardation.
