# Supplementary material for: A Rare Cause of Chronic Hypokalemia with Metabolic Alkalosis: Case Report and Differential Diagnosis
Source: Children (Basel). 2020 Nov 5;7(11):212. doi: 10.3390/children7110212 (PMC7694404; doi:10.3390/children7110212)
Supplement: Supplementary file 1 [file children-07-00212-s001.pdf]

Supplementary Table 1. Criteria for Classifying Pathogenic Variants according to ACMG guidelines*.

Evidence of pathogenicity	Category
**Very strong**	**PVS1** Null variant (nonsense, frameshift, canonical +/−1 or 2 splice sites, initiation codon, single or multi-exon deletion) in a gene where loss of function (LOF) is a known mechanism of disease
**Strong**	**PS1** Same amino acid change as a previously established pathogenic variant regardless of nucleotide change
	**PS2** De novo (both maternity and paternity confirmed) in a patient with the disease and no family history
	**PS3** Well-established in vitro or in vivo functional studies supportive of a damaging effect on the gene or gene product
	**PS4** The prevalence of the variant in affected individuals is significantly increased compared to the prevalence in controls
**Moderate**	**PM1** Located in a mutational hot spot and/or critical and well-established functional domain (e.g. active site of an enzyme) without benign variation
	**PM2** Absent from controls (or at extremely low frequency if recessive) in general population databases #
	**PM3** For recessive disorders, detected in trans with a pathogenic variant. This requires testing of parents (or offspring) to determine phase
	**PM4** Protein length changes due to in-frame deletions/insertions in a non-repeat region or stop-loss variants
	**PM5** Novel missense change at an amino acid residue where a different missense change determined to be pathogenic has been seen before
	**PM6** Assumed de novo, but without confirmation of paternity and maternity
**Supporting**	**PP1** Co-segregation with disease in multiple affected family members in a gene definitively known to cause the disease
	**PP2** Missense variant in a gene that has a low rate of benign missense variation and where missense variants are a common mechanism of disease
	**PP3** Multiple lines of computational evidence support a deleterious effect on the gene or gene product (conservation, evolutionary, splicing impact, etc)
	**PP4** Patient's phenotype or family history is highly specific for a disease with a single genetic etiology
	**PP5** Reputable source recently reports variant as pathogenic but the evidence is not available to the laboratory to perform an independent evaluation

The criteria selected to classify the variant c.900dup, p.(Glu301Argfs*56) as pathogenic are shown in green. * Richards S, Aziz N, Bale S, et al. Standards and guidelines for the interpretation of sequence variants: a joint consensus recommendation of the American College of Medical Genetics and Genomics and the Association for Molecular Pathology. Genet Med. 2015;17(5):405–424.). # the variant c.900dup, p.(Glu301Argfs*56) has not been described in gnomAD database (https://gnomad.broadinstitute.org/).
